# Health and Care Workers in Pandemic Recovery: Major Challenges and Solutions

**DOI:** 10.34172/ijhpm.2023.8081

**Published:** 2023-05-07

**Authors:** Viroj Tangcharoensathien, Ibadat Dhillon

**Affiliations:** ^1^International Health Policy Program, Ministry of Public Health, Nonthaburi, Thailand; ^2^World Health Organization, South-East Asia Regional Office, New Delhi, India

## Dear Editor,

 Community health workers (CHWs) are defined as paraprofessionals or lay individuals with an understanding of local culture and language, have received short duration standardised job-related training; their primary goal is to provide culturally appropriate health services to the community.^1,2^ CHWs enable the health system to tap into the social capital that exists in rural and urban communities.^3^

 Two recent papers published in *International Journal of Health Policy and Management *highlighted the important role and contributions of CHWs, using the case of Accredited Social Health Activist (ASHA) in India by Kane et al^4^ and volunteers supporting hospice and palliative care during COVID-19 by Walshe et al.^5^ Findings on ASHA confirm prior systematic reviews which suggest that CHWs with full support can minimize equity gaps in marginalized communities.^6^ A study emphasises the need for greater focus on CHW programme governance, including community engagement in selection, community voice and collaboration with CHWs through local bodies.^7^ Often ASHAs have to strike a balance between expectations by their families and self-interest, health staffs they work with and the communities’ expectation. Competing interests sometimes result in ASHA maximizing their own benefit. Therefore, it is essential that ownership and accountability rest firmly with the communities they serve. Ownership by the community, supportive supervision, continuous education, adequate logistical are enabling factors for CHW contributions.^8^ CHWs in the United States demonstrates good outcome and positive return on investment.^9^ Volunteers also contributed to pandemic responses.^10-12^

 Double shocks (health and economic) posed by COVID-19 pandemic and subsequent economic challenges notably fiscal crunch complicated by high debt servicing have had major impacts on all countries though unevenly. With an uneven economic recovery,^13^ some low- and lower-middle income countries are struggling to mobilize domestic resources to scale up COVID-19 vaccination and ensure health systems recovery.^14^

 The COVID-19 pandemic has posed further challenges on the international migration of health workers. As illustration, over the last decade (2012 to 2021) the United Kingdom saw the doubling of the annual inflow of foreign-trained doctors and trippling for foreign-trained nurses. Such increases were particularly notable in 2021. The UK General Medical Council reported that 7377 (37%) of the 19 977 doctors who started work in the National Health Service in 2021 had a British qualification; the rest were foreign-trained doctors.^15^

 Across the Organisation for Economic Co-operation and Development (OECD) countries, stock and annual inflow of foreign-trained doctors and nurses are at record levels during pandemic.^16^ Interpretation of OECD statistics needs cautions, as international migration was not homogeneous, though high flows were reported by the United States and the United Kingdom. The World Health Organization (WHO) reported that compared with pre-COVID-19, in 2021 there was 27% increase in annual inflow of foreign trained medical doctors in 17 OECD countries, and a 79% increase in annual inflow of foreign trained nurses in 15 OECD countries. Given the administrative and regulatory flexibilities for health professionals in high-income countries, we foresee higher international migration of health workers than other workers.

 Both push and pull forces spur the international migration of health personnel. A recent study from the United Kingdom highlighted that the factors driving doctors’ immigration to the United Kingdom were the same as those driving doctors’ emigration out of the United Kingdom: poor working conditions, employment opportunities, better training and career advancement opportunities, better quality of life, desire for a life change and financial reasons.^17^

 Low- and middle-income countries are especially challenged in retaining their health workers. Indeed, WHO reported 55 countries are in critical shortage of health workforce to be able to achieve Sustainable Development Goal by 2030. These countries belong to support and safeguard list, based on a density of doctors, nurses and midwives below the global median of 49 per 10 000 population and a universal health coverage service coverage index below 55.^18^ Though the list does not prohibit international recruitment, governments are informed about the negative impact on the health system in these countries. Many high-income country governments adhere to these agreements, private recruiters have yet to do so.

 Health systems recovery from pandemic requires governments in all countries, rich or poor, increase investment in education, deployment, retention and improve work conditions, provide occupational safety and adequate payroll of health workers, while in parallel boost the contributions by CHW and volunteers to health systems.

## Ethical issues

 Not applicable.

## Competing interests

 Authors declare that they have no competing interests.

## Disclaimer

 The work represents the personal opinion of the author (Ibadat Dhillon) and not that of the organization for whom they work.
